# Progress in Avian Anatomy for the Years 1888-1889

**Published:** 1890-01

**Authors:** R. W. Shufeldt


### PROGRESS IN AVIAN ANATOMY FOR THE YEARS 1888–1889.[1]

BY R. W. SHUFELDT, MD., C.M.Z.S.

IN reviewing the progress made in the anatomy of birds during the past year, (Nov. 1888, to Nov. 1889), it will be seen that no very extensive single work upon the subject has appeared since the last Congress of this Union.

Nevertheless, it is gratifying to know that the interest evinced in the study of the structure of the group has been steadily on the increase in ornithological quarters everywhere, and although the published work in these fields during the twelvemonth just past, has not been voluminous, even this fact may portend a calm before the storm, and ere the time comes round for our next report, another epoch-making volume may be sent forth. It is not every year that sees such a work that the distinguished Dutch anatomist, Max Fürbringer, gave to the science in 1888. Nor is the day anywhere in the near future that will see a diminution of the influence that that classic monument is to have upon the general science of ornithology.

Whenever I have anything to say in reference to the progress of the study of the structures of birds, my mind first reverts to England, a country that has so long been characterized as a cen-

[1] Read at the Seventh Congress of the American Ornithologists' Union (held in New York, November 12, 1889, and following days) by the writer, it being his Report to the Union as Secretary of the Committee on Avian Anatomy.

tre of activity in such matters. From there we have received from the pen of William Kitchen Parker several essays that are to be added to the long list of memoirs in avian morphology of that tireless worker. Among these we have for the first time a full account of the skeleton of *Steatornis caripensis*, handsomely illustrated with plates; and in it Prof. Parker goes to show how that interesting form is structurally linked to others of its kind. Other types have also received his notice, as the Flamingoes, the *Limicolæ*, and *Opisthocomus*. Perhaps I may be pardoned, however, for a slight breach of confidence when I say that the work done, the great labor accomplished in bird anatomy during the past year by Prof. Parker, will come in at some future report; and this likewise applies to similar labors that have been under the hand of his distinguished son of the Otago Museum in New Zealand.

Prof. Hans Gadow is still bringing out in Bronn's "Thierreichs" his admirable chapters on the study of the structure of birds in general, and during the past twelvemonth this able anatomist has been principally engaged upon the viscera of the Class, and I am informed that some of that work will soon appear.

Of equal value have been some of the several productions of Beddard in the same fields, and among these I may mention his paper "On Certain Points in the Anatomy of the Accipitres with Reference to the Affinities of *Polyboroides*," which came out in February of this year in the Proceedings of the Zoölogical Society of London, where he so ably conducts the prosectorial department.

In France there has appeared the "*Traité d'Ostéologie Comparée*" of Pouchet and Beauregard, which has in it a chapter devoted to the osteology of birds, the best one, in a work that throughout has perhaps adhered too closely to the Cuvierian and older systems of classification.

A Florentine naturalist of note, Sig. Ettore Regalia, has published a short but interesting paper presenting a list of those birds in the avifauna of Italy that possess a claw upon the terminal phalanges of the second and pollex digits.

To all these might be added the many other essays on the same topics, coming from the pens of numerous writers of several of the countries on the continent, but their consideration would carry the writer beyond the limits that he has set upon this report.

Turning to our own country we are to notice the excellent papers upon tne anatomy of birds that have been published during the past year by a worthy associate member of this Union, Mr. F. A. Lucas of the U. S. National Museum. In a paper that appeared in *The Auk* on "The Main Divisions of the Swifts," he established the new Family *Dendrochelidonidæ*, while in the publications of the U. S. National Museum appeared an excellent essay devoted to "Notes on the Osteology of the Thrushes, *Miminæ* and Wrens," and finally, an article giving a "Description of Some Bones of Pallas's Comorant," wherein the affinities of that bird and the red-faced Comorant are shown, as well as the distinction between them and *P. carbo* and *P. dilophus*.

Mr. Lucas during the year has also given considerable attention to the study of the skeleton of the Great Auk, basing his researches upon the unrivalled collection of the remains of that bird, in the U. S. National Museum, a collection made by himself some time ago at one of the old haunts of that now extinct type, in the St. Lawrence River. I understand that this latter work is through the press and may be looked for at an early day.

It will be a great pleasure to many to know, as it was to myself, that there will soon appear from the presses of the most celebrated publishing house in Europe, a volume that will reproduce "Field Ornithology and the General Part" of Dr. Coues's "Key" to the N. American birds. And when one thinks for a moment of the far-reaching influence, and a most beneficial influence, that the anatomical part of that work has had in making clear to the hundreds of students in ornithology, the elementary steps in bird structure, the fact can only be dwelt upon with extreme gratification.

Since the last Congress of the Union, the writer's attention has been mainly devoted to the production of a work having to do with the anatomy of certain Reptiles,—and Birds, I fear, at my hand have had rather the go by for the year past. However, some work has been accomplished by the Secretary of your Committee, and such as it is, it is here recapitulated to form a part of this report.

In the November, 1888, isssue of *The Journal of Morphology*, under the title "On the Affinities of *Aphriza Virgata*," I published a short paper of about thirty pages, illustrated by a plate, giving a complete account of the skeleton of that form, and comparing it with the Turn-stones, Oyster-catchers and others. Early in the spring of this year, the U. S. National Museum published two other papers of mine, one, the "Observations upon the Osteology of the North American *Anseres*," a thirty-page 8vo. paper with 30 figures in its text ;the other the "Observations upon the Osteology of the Orders *Tubinares* and *Steganopodes*" a sixty-two 8vo. paper with 43 figures illustrating its subject. During the year there has also appeared of mine, four parts of the "Contributions to the Comparative Osteology of Arctic and Subarctic Water-Birds, in the *Journal of Anatomy* of London. These four parts comprised some 125 pp. 8vo. with over 50 figures devoted to the illustration of the skeletons of our Arctic water-fowl. At about the same time, or in April, 1889, THE JOURNAL OF COMPARATIVE MEDICINE AND SURGERY published for me a memoir of some twenty-five pages, with 17 figures in the text, entitled "Osteology of *Circus hudsonius*," an article which presents a complete account of the skeleton in the Marsh Hawk.

A littte later, the same journal kindly printed for me in two parts my "Osteological Studies of the Sub-family *Ardeinæ*," the two comprising a paper of some fifty-five pages, and between thirty-five and forty figures in the text. The first of these parts appeared in July, 1889, the last in October of the same year, that is the first of last month. Prior to this, however, or in June, 1889, *The Journal of Morphology*, published two other papers of mine, the one on "Contributions to the Comparative Osteology of the Families of North American *Passeres*," appeared as a memoir of some thirty-one pages, illustrated by twenty-six figures on stone ; the remaining essay was a short one of about ten pages with a Plate, and entitled "Notes on the Anatomy of *Speotyto cunicularia hypogæa*." All of the above essays are thoroughly comparative in their treatment, based upon abundant material, but my space will obviously preclude my entering upon any of their contents in detail.

A more extended article than any of the foregoing was a memoir of some hundred pages, and eight lithographic plates, devoted to the comparative morphology of the Swifts, Humming-birds and Goatsuckers, which was with great generosity brought out for me by the Linnæan Society of London, about the first of last September.

It will thus be seen that the Committee can report the publication of a work, in the aggregate, on the anatomy of our United

States birds, of some 468 pages, and illustrated by upwards of 267 original figures.

By some it will be remembered that Dr. Coues, the Chairman of this Committee, kindly read a paper of mine at the last Congress of the Union, entitled "On the Position of *Chamæa* in the System." The writer has completely revised that memoir, largely added to it, through the description of additional material, and it will now shortly appear in print.

Very frequently during my labors upon the anatomy of the Class, I have greatly felt the need of a good hand-book to the muscles of birds, and in looking about me, I soon found that we possessed no such manual in the English language, at least the kind of work that the thorough dissector required. To meet this want I undertook the preparation of a volume devoted to that subject. A thoroughly cosmopolitan form, or rather a form well representing a cosmopolitan group of birds, the Raven, was selected. I carefully dissected out on many specimens every muscle of this type, and figured them in a careful series of drawings. These I supplemented by a series of drawings of the skeleton of the same form, and on the bones indicated the origin and insertion of all the muscles. Full descriptions were written out, and the groups of muscles classified ; and finally some comparative work was added. Both the drawings of the muscular system, as well as the skeleton are life size, which make the parts very clear and convenient for use. To my surprise, when it was all completed, the MSS. for a small volume were on my hands. This has now been accepted by Messrs. Macmillan & Company of London and New York, for publication, and went to press about the middle of last month. And your Committee trust that before another Congress of this Union convenes they will be enabled to place a copy of this latter work before you.

